# Psychometric properties of the Hungarian version of the ages and stages questionnaires: Social–emotional‐2 for 18‐month‐old children

**DOI:** 10.1002/imhj.70050

**Published:** 2025-10-09

**Authors:** Melinda Pohárnok, Beatrix Lábadi, Eszter Regőczi‐Balogh, Krisztina Kopcsó

**Affiliations:** ^1^ Department of Developmental and Clinical Psychology University of Pécs Pécs Hungary; ^2^ Department of General and Evolutionary Psychology University of Pécs Pécs Hungary; ^3^ Hungarian Demographic Research Institute Budapest Hungary

**Keywords:** ages & stages questionnaires: social‐emotional‐2 (ASQ:SE‐2), early screening, reliability, representative sample, social‐emotional development, validity

## Abstract

In Hungary there is no standardized tool to screen early social‐emotional difficulties. This study aimed to evaluate the psychometric properties of the Hungarian version of the Ages and Stages Questionnaires: Social‐Emotional Second Edition for 18‐month‐olds (ASQ:SE‐2/18). Two studies were conducted. Study 1 involved translation, cultural adaptation, and validation in a convenient sample (*N* = 423). Study 2 used a nationally representative sample (*N* = 4918) to evaluate the factor structure, internal consistency, predictive validity and socioeconomic and demographic correlates. Study 1 supported the cultural adequacy of the Hungarian ASQ:SE‐2/18, and a comparison with the Child Behavior Checklist 1.5–5 provided evidence for its validity. In Study 2, the two‐factor model, consisting of Emotional Difficulty (*α* = .74) and Social Difficulty (*α* = .69), fit better than the single‐factor model and showed acceptable internal consistency. Being at risk for social (odds ratio = 1.7) or emotional (odds ratio = 3.6) development at 18 months predicted socio‐emotional difficulties at age 3 assessed by the Strengths and Difficulties Questionnaire. Socioeconomic disadvantages were correlated with higher levels of social‐emotional risk. In conclusion, the study demonstrated the reliability of the Hungarian ASQ:SE‐2/18 and offered evidence supporting its validity. Emotional difficulties at 18 months strongly predict later maladjustment, emphasizing the need for early screening and further tool development.

## 1 INTRODUCTION

In 2019, 2% of European children under the age of 5 years suffered from a mental health condition, and the prevalence of mental health problems was as high as 10% among children aged between 5 and 9 years (UNICEF, 2024). Hungarian general practitioner reports submitted to the Hungarian Central Statistical Office disclosed that, 3‐5% of children aged 5–14 years were diagnosed with a mental or behavioral health condition in Hungary between 2017 and 2019 (Hajdu & Kertesi, 2023). This suggests that mental health problems and behavioral difficulties in the early years affect many children in the European region and in Hungary.

Prior research has shown that early childhood delays or deficits in competencies and behavioral problems predicted school‐age teacher reported behavior problems and parent‐reported psychiatric disorders (Briggs‐Gowan & Carter, 2008). Furthermore, externalizing problems at 2 years of age demonstrated strong predictive power for externalizing problems at 5 years of age (Mäntymaa et al., 2012), while severe difficulties of communication and interaction in late infancy predicted communication and interaction problems in the early preschool period (Möricke et al., 2014).

The relatively high prevalence of mental and behavioral disorders throughout childhood, and the evidence supporting the temporal stability of such problems from toddlerhood through middle childhood draw attention to the importance of early screening. Earlier identification of risks leads to more cost‐effective and efficient interventions, and the early recognition of red flags for delayed or impaired development is essential for both mental (Colizzi et al., 2020) and neurodevelopmental (Micai et al., 2020) disorders.

### 1.1 Early screening in Hungary

In Hungary, upon confirmation of pregnancy, a pregnant woman is required to contact her district health visitor who provides primary health care for both the mother and the child until the child starts school (Szabó et al., 2021). During early childhood, health visitors carry out comprehensive (parent‐completed) developmental screening tests at 1, 2, 4, 6, 9, 12, 15, 18, and 24 months then annually until the child reaches 6 years of age (Altorjai et al., 2015). These 12‐item tests cover six areas: language and speech development, psychomotor (e.g. fine motor skills) and mental development, social development and behavior problems, motor (e.g. gross motor skills) and sensory development. If a dysfunction or developmental problem is identified, the health visitor is required to direct the family to the pediatrician or general practitioner who is authorized to refer the child to specialists.

**Key Findings**

- There is evidence of validity for the Hungarian adaptation of ASQ:SE‐2/18.
- Disadvantaged socioeconomic background hindered social‐emotional development.
- Risk identified at 18 months predicted social‐emotional difficulties at 36 months.

**Statement of the relevance of the work for infant and early childhood mental health**

Early identification of social‐emotional difficulties is crucial for early childhood intervention, particularly among disadvantaged populations. Culturally valid measures suitable for use in primary health care are needed to screen for early social‐emotional risks. Furthermore, it is important to accurately identify developmental risk behaviors that may predict later behavioral problems.

However, comparing the rate at which health visitors identify children as having significant social, mental, motor, or behavioral delays or risks to screening rates in other countries questions the effectiveness of the health visitor screening tool. In Hungary, 1%–7% of children aged 12–36 months are classified as having significant emotional or behavioral delays or risks (Hajdu & Kertesi, 2023). Whereas studies applying the most widely used assessment tools have shown higher prevalence rates worldwide: 5%–9.4% measured by the Child Behavior Checklist (CBCL, Achenbach & Rescorla, 2000) in a German sample of 2.5–5‐year‐olds (Hahlweg et al., 2008); 19.4% measured by the Brief Infant‐Toddler Social and Emotional Assessment (BITSEA, Briggs‐Gowan et al., 2004) in a U.S. sample of children aged 18–36 months (Gleason et al., 2010); and 8.5%–14.9% measured by the Strengths and Difficulties Questionnaire (SDQ, Goodman, 1997) in a Swedish sample of children aged 13–71 months (Gustafsson et al., 2017). The presumably low detection rate of the health visitor screening makes it crucial to adopt an early infant/toddler screening tool in Hungary that provides a comprehensive and more sensitive parent‐reported assessment, specifically for socio‐emotional development.

### 1.2 Screening social‐emotional development by ASQ:SE‐2

The Ages and Stages Questionnaires: Social‐Emotional Second Edition (ASQ:SE‐2) is a series of parent‐completed screening questionnaires for children aged 1–72 months, the revised version of the Ages and Stages Questionnaires: Social‐Emotional (ASQ:SE; Squires et al., 2002). The questionnaires focus on seven behavioral aspects of early childhood mental health and social‐emotional competence: self‐regulation, compliance, communication, adaptive behaviors, autonomy, affect, and interactions with people. The purpose of the questionnaires is to identify risks or delays in socio‐emotional development. Based upon their total score children can be categorized into no risk, moderate risk (monitoring zone), or high risk (referral for further assessment is needed) groups (Squires et al., 2015). The monitoring zone is, however, primarily for use by practitioners, as it indicates that the child's development should be followed more closely. Consequently, adaptation studies of the ASQ:SE and ASQ:SE‐2 questionnaires (Anunciação et al., 2019; Alvarez‐Nuñez et al., 2020; Xie et al., 2022) typically differentiate between children at risk (i.e. high risk, with a need for a referral for further assessment and/or intervention services) and those not at risk.

The validity and reliability of the ASQ:SE have been extensively tested in international representative samples mainly throughout Asia, the Americas and Western‐Europe, and the measure has been used in Head Start, Early Head Start, home visiting, and prekindergarten programs, across the United States (for a review, see Chen et al., 2020). In recent years, the psychometric properties of the ASQ:SE‐2 have also been investigated worldwide (e.g. in Taiwan, Chen et al., 2019; in China, Xie et al., 2022, in Sweden, Edenius et al., 2024; and in Iran, Shariatpanahi et al., 2024). These studies have found that the ASQ:SE‐2 possessed adequate psychometric properties for detecting early social‐emotional risks.

Most studies investigating the construct validity and dimensionality of ASQ:SE or ASQ:SE‐2, concluded that the two‐factor model proposed by Chen et al. (2016) showed the best fit across most age intervals (Chen et al., 2020), across some age intervals (Anunciação et al., 2019; Xie et al., 2022) or, at least, for younger age groups (6‐, 18‐ and 24 months; Alvarez‐Nuñez et al., 2020). In this model, items are categorized into social competence and emotional competence factors. Social competence includes skills related to adaptive communication with peers and caregivers, as well as expression of positive affection and affiliation. Emotional competence refers to physiological and emotion regulation. For the 18‐month‐old age group, the factor analytic studies of Chen et al. (2016) and Alvarez‐Nuñez et al. (2020) found sufficient model fit for the two‐dimensional solution of ASQ:SE. Edenius et al. (2024), however, suggested a one‐factor solution for the 18‐month ASQ:SE‐2, albeit with a reduced set of items.

Studies reporting the reliability of the 18‐month version of the ASQ:SE or ASQ:SE‐2, found that the internal consistency of the total scale (as measured by Cronbach's alpha or McDonald's omega) ranged from excellent (.85, Alvarez‐Nuñez et al., 2020, ASQ:SE; .91, Squires et al., 2015, ASQ:SE‐2) to poor (.54, Shariatpanahi et al., 2024, ASQ:SE‐2). Alvarez‐Nuñez et al. (2020) also investigated the internal consistency of the two previously validated subscales and found them to be appropriate (.84 for the Social competence and .74 for the Emotion competence subscale).

As there is ample evidence that children's early social‐emotional development is related to their sociodemographic background and their parents’ mental health (Masarik & Conger, 2017), several international adaptation studies have investigated these relationships. Regarding gender, studies generally have indicated that a higher percentage of boys are at risk across all age ranges (Alvarez‐Nuñez et al., 2020; Kucuker et al., 2011; Squires et al., 2004) with gender differences becoming more expressed in preschool age groups compared to infancy and toddlerhood (Chen et al., 2015). Regarding socioeconomic and maternal factors, research has shown that early social‐emotional developmental risk is associated with lower household income (Alvarez‐Nuñez et al., 2020; Vaezghasemi et al., 2023), low parental education (Vaezghasemi et al., 2023) and poorer maternal mental health (Agarwal et al., 2024; Vaezghasemi et al., 2023).

## 2 THE CURRENT STUDY

The presumably low detection rate of early social, emotional or behavioral problems in the Hungarian health visitor screening and the significant social inequalities in children's mental health (Hajdu & Kertesi, 2023) underscores the need to adapt a reliable and valid screening tool for early social‐emotional risks. The ASQ:SE‐2 is an internationally recognized, easy‐to‐use, parent completed questionnaire, that may be suitable for both research purposes and for integration into the health visitor system. Therefore, the aim of our research was to adapt the 18‐month ASQ:SE‐2 questionnaire (ASQ:SE‐2/18) into Hungarian and to evaluate its applicability. The choice of the 18‐month version was driven by two reasons. First, a methodological constraint: the waves of the birth cohort study, which constituted the nationally representative sample, were pre‐planned to include an 18‐month assessment (for further details see Study 2). Second, the 15–21‐month age range covered by the ASQ:SE‐2/18 precedes the typical age at which children enter formal childcare settings, where developmental monitoring becomes more systematic. Therefore, this tool can be used to detect early red flags of later emotional and behavioral problems of Hungarian children before they enter institutional care, typically at ages two or three.

This study presents two consecutive research in which the reliability and validity of the Hungarian version of the ASQ:SE‐2/18 were examined in two steps. In the first study, we conducted the linguistic and cultural adaptation of the questionnaire. In the second study we carried out a large‐scale empirical study to assess the psychometric properties of the scale, including construct validity and internal consistency. Our findings contribute to the early identification of social‐emotional developmental difficulties in Hungary and support the refinement of screening procedures and preventive interventions in the region.

## 3 STUDY 1

### 3.1 The aim of Study 1

Study 1 had two objectives. The first was to conduct the translation and cultural adaptation of the Ages and Stages Questionnaire: Social‐Emotional‐2 developed for 18‐month‐old children (ASQ:SE‐2/18). The second was to investigate the appropriateness of the Hungarian version of the ASQ:SE‐2/18. Therefore, we tested the internal consistency and the convergent validity of the tool by investigating its specificity and sensitivity. We established a tentative cut‐off score, intended as a suggested threshold for further investigation and validation in the second study.

### 3.2 Method

#### 3.2.1 Participants

Study 1 used convenience sampling. Parents of children between the ages of 15 months 0 days and 20 months 31 days (adjusted age for prematurity) participated in this study. Participants raising a child outside this age range or with missing data on the ASQ:SE‐2 were excluded. In the final total sample, 423 caregivers of children (52.5% males) participated. The total sample included a subsample of 100 children (45.0% males) with higher proportion at elevated developmental risk. In this subsample, alongside the ASQ:SE‐2/18, we also administered the CBCL 1.5–5 for examining convergent validity. In the total sample, 9.5% of children were born preterm, while in the subsample this proportion was 40%. Preterm children's age was corrected for number of weeks they were born prematurely. Descriptive characteristics of the participants are presented in Table 1.

**TABLE 1:** Participants’ characteristics for the total sample and the validity measure subsample.

	Total sample^a^ (*n* = 423)	Subsample (*n* = 100)
Age in months		
*M* (SD)	17.3 (1.7)	17.2 (1.9)
Sex		
Male, *n* (%)	222 (52.5%)	45 (45.0%)
Female, *n* (%)	201 (47.5%)	55 (55.0%)
Gestational age in weeks, *M* (SD)	–^b^	36.0 (4.1)
Gestational weight in grams, *M* (SD)	–^b^	2687.2 (975.5)
Preterm birth, *n* (%)	40 (9.5%)	40 (40.0%)
Mother's educational attainment		
Low, *n* (%)	6 (1.4%)	2 (2.0%)
Medium, *n* (%)	167 (39.5%)	31 (31.0%)
High, *n* (%)	248 (58.8%)	67 (67.0%)
Mother's age in years		
*M* (SD)	32.4 (4.7)	33.3 (5.4)
Range	21–46	23–46

^a^ Total sample included the subsample.

^b^ The gestational age and weight were assessed only in the subsample.

#### 3.2.2 Procedure

Primary caregivers were recruited via social media and parenting websites. To assess convergent validity of ASQ:SE‐2/18, we intentionally included a higher proportion of preterm children in the subsample, given their increased risk of social‐emotional difficulties. As a result, the advertisement was also shared on platforms specifically targeting parents of preterm children. Caregivers completed an online survey including demographic questions, ASQ:SE‐2/18, and Child Behavior Checklist (CBCL 1.5–5) (CBCL was completed only by the caregivers in the subsample).

Participation was voluntary in this study, with written consent obtained on the first page of the survey. The study was conducted in accordance with the Helsinki Declaration and Code of Ethics of Hungarian Psychological Association. The study was approved by an Ethical Committee (reference number 2022/1) set up for the Cohort ’18 study, which included professionals who were not part of the study group.

#### 3.2.3 Measures

##### 3.2.3.1 Demographic information

The demographic questions asked caregivers to provide information on the child's sex, age in months and gestational age in weeks. Information about caregiver's age in years, education level was also requested. The caregiver's education level was coded based on the International Standard Classification of Education 2011 (UNESCO Institute for Statistics, 2012) into three categories: low (ISCED 0‐2), medium (ISCED 3‐4) and high (ISCED 5‐8).

##### 3.2.3.2 Ages and Stages Questionnaire: Social Emotional‐2

The ASQ:SE‐2 consists of a series of questionnaires designed for completed by a child's primary caregiver. It consists of nine questionnaires, covering nine intervals for children aged 1–72 months: the 2‐, 6‐, 12‐, 18‐, 24‐, 30‐, 36‐, 48‐, and 60‐month versions. Each questionnaire focuses on social and emotional areas, with the number of items increasing as the child grows older (from 19 items on the 2‐month interval to 33 items on the 48‐ and 60‐month intervals). Parents or caregivers rate their child's behavior on a 3‐point scale, whether the child does the behavior, with scoring depending on whether the items relate to competencies (often or always = 0; sometimes = 5; rarely or never = 10) or difficulties (often or always = 10; sometimes = 5; rarely or never = 0). Caregivers could also indicate if they are concerned about the child's behavior; in that case, an additional 5 points are added to the total score. Higher total scores indicate potential problems and lower scores suggest competent social and emotional behavior. The ASQ:SE‐2 questionnaire also includes explanatory questions, but these were not administered in this study.

In our study we used the questionnaire developed for 18‐month‐old children. It contains 31 items. The total score is calculated as the sum of the parent's responses, with a range from 0 to 465 points. As a screening tool, children scoring above the established cut‐off scores are considered “at risk” and are recommended for further evaluation. The questionnaire includes three additional overall items addressing maternal concerns about eating and sleeping behaviors, as well as general worries; however, scores are not calculated for these items.

##### 3.2.3.3 The Hungarian translation of ASQ:SE‐2/18

The Hungarian adaptation of the ASQ:SE‐2/18 followed the procedures outlined in the Guidelines for Cultural and Linguistic Adaptation of ASQ‐3 and ASQ:SE (Squires et al., 2013). Initially, permission for the adaptation and translation into Hungarian was obtained from Brooks Publishing. The ASQ:SE‐2/18 was then translated from English into Hungarian by two independent translators with expertise in early childhood development. Then the two Hungarian translations were compared by the research team, with the linguistically most appropriate version selected in cases of discrepancy. Upon completing the translation process, pilot testing was conducted with caregivers through a focus group (*N* = 6). Caregivers’ feedback was evaluated to ensure that the items were clear, culturally appropriate, and free of ambiguity. Subsequently, an independent translator, who was unfamiliar with ASQ:SE‐2/18, performed a back‐translation into English. The research team compared the back‐translation with the original English version to identify and correct any discrepancies. Finally, the Hungarian translation and its English back‐translation were submitted to the publishing company for verification and approval. Upon approval, the final version of the ASQ:SE‐2/18 translation was then completed and prepared for general use.

##### 3.2.3.4 Child Behavior Checklist 1.5–5 years (CBCL 1.5–5)

The 1.5–5 years old version of the Child Behavior Checklist (CBCL 1.5–5; Achenbach & Rescorla, 2000) was used in our study to assess the convergent validity of ASQ:SE‐2/18. We used the Hungarian adaptation of the questionnaire which was carried out within the framework of an Economic Development and Innovation Operational Program (GINOP) research project (GINOP‐2.1.7‐15‐2016‐0,1561) and is available through ’Ask For’ Ltd. The CBCL 1.5–5 assess emotional, behavioral and social problems in early childhood. The instrument includes a total of 99 items, each rated on a 3‐point scale (0 = not true as far as I know; 1 = somewhat or sometimes true; 2 = very true or often true). The caregiver ratings were completed following the standardized instructions outlined in the manual by Achenbach and Rescorla (2000). CBCL 1.5–5 is a well‐standardized and validated questionnaire with strong internal consistency. In our study, the internal consistency of the instrument was excellent (Cronbach's *α* = .92 for Total Problems, Cronbach's *α* = .84 for externalizing problems and Cronbach's *α* = .78 for internalizing problems). T‐scores for externalizing, internalizing and total problem scales were used to indicate the degree to which child deviates from normative criteria. T‐scores below 60 were interpreted as falling within the normative range of development, whereas children with T‐scores of 60 or above were considered indicative of potential behavioral or emotional problem (with a risk range of 60–64, and clinical symptoms above 64) (Achenbach & Rescorla, 2000). For statistical analyses, T‐scores for externalizing problems and internalizing problems and total problems were used to determine the developmental risk.

### 3.3 Data analyses

JAMOVI 2.3 software (2022) was used to analyze the data. Descriptive statistics were calculated for demographic characteristics and for the ASQ:SE‐2/18 total score (means, standard deviations, medians, and semi‐interquartile ranges). Cronbach's alpha was calculated to test internal consistency for the entire sample. We used Mann‐Whitney U test to examine the gender differences for ASQ:SE‐2/18.

Two approaches were used to establish a tentative cut‐off score. First approach followed the guidelines of Squires et al. (2015), which recommended using the semi‐interquartile range and medians instead of mean scores and standard deviations when calculating cut‐off scores. This method is advantageous because scores from most screening tests are typically not normally distributed and tend to be positively skewed in healthy population. The skewness is largely due to the high proportion of typically developing children in a normative sample, whose score falls within the low range, indicating few problem behaviors. Consequently, we calculated the cut‐off score by adding 1.5 semi‐interquartile range to the medians.

Second approach used Receiver Operating Characteristic (ROC) analysis to determine the cut‐off score by comparing children's data on ASQ:SE‐2/18 with their classification (children with behavioral and emotional problems [hereafter labelled as at risk] vs. typically developing) on the convergent instrument CBCL 1.5–5. ROC curves were computed using ASQ:SE‐2/18 total score and a dichotomous CBCL 1.5–5 variable as the criterion (at risk vs. typically developing). The CBCL 1.5–5 dichotomous variable was determined using T‐scores from Externalizing and Internalizing scales; children with scores of 60 or above on either scale were classified as at risk. The Area Under Curve (AUC) was computed with an AUC of .50–.70 reflecting low accuracy, .70–.90 moderate accuracy and .90–1.00 high accuracy (Youden, 1950; Fischer et al., 2003). Children were classified into four groups: (1) true positive: problem or risk on both tools; (2) true negative: no problem or risk on both tools; (3) false positive: risk on ASQ:SE‐2, but no problem or risk on CBCL 1.5–5; (4) false negative: no risk on ASQ:SE‐2, but risk or problem on CBCL 1.5–5. Then specificity and sensitivity values were calculated (≥70 % were considered good). Finally, we tested the correlations (Spearman's *rho*) between CBCL 1.5–5 subscales and ASQ:SE‐2/18.

### 3.4 Results

#### 3.4.1 Hungarian adaptation

Based on the translation and back‐translation process, and the feedback from the parental focus group, only minor adjustments were made to tailor the questionnaire to Hungarian. Consequently, cultural modifications of the Hungarian adaptation were minimal, ensuring equivalence with the original version. These included replacing the informal term “baby” with “child” for consistency and omitting gender references due to the lack of gender differentiation in Hungarian personal pronouns. We modified one example in item 28 – substituting “*fly a toy airplane*” with “*pretends to drive a car*”, because children are generally more familiar with the act of pretending to drive a car than flying a toy airplane, making the former a more accessible and relatable form of symbolic play. Brooks Publishing has accepted our modification for the translation.

#### 3.4.2 Descriptive statistics

Descriptive statistics including means, standard deviations, medians and semi‐interquartile ranges were computed for ASQ:SE‐2/18 total score (see Table 2). The scores did not exhibit normal distributions (Shapiro–Wilk *W *= .897, *p *< .001).

**TABLE 2:** Descriptive statistics of the ASQ:SE‐2/18 total scores for the total sample and the subsample.

	ASQ: SE‐2/18 total sample (*n* = 423)	ASQ: SE‐2/18 subsample (*n* = 100)
*M* (SD)	32.1 (21.1)	35.1 (26.4)
Median	30	30
Semi‐interquartile range	10	15
Range	0–150	0–150
90^th^ percentile	55	70.5
Skewness (se)	1.54 (.11)	1.55 (.24)
Kurtosis (se)	4.25 (.23)	3.40 (.47)
Risk % (cut‐off ≥ 40)^a^	23.4%	26.0%
Risk % (cut‐off > 45)^b^	17.7%	24.0%

^a^ The cut‐off point of ≥40 was determined through a ROC analysis performed on the subsample.

^b^ The cut‐off point of >45 was calculated based on the 1.5 semi‐interquartile range and median, as suggested by Squires et al. (2015).

Comparing the ASQ:SE‐2/18 scores between males and females, no difference was found (*Mdn *= 30 for both sexes, *U *= 21,595, *p *= .62).

#### 3.4.3 Internal consistency

Cronbach's alpha coefficient was .68 for the total sample and was .77 for the subsample.

#### 3.4.4 Convergent validity assessment

To establish a tentative cut‐off value for ASQ:SE‐2/18 we used two approaches. First, following Squires et al. (2015), the cut‐off score was calculated by adding 1.5 semi‐interquartile range to the median. Using this method, the cut‐off score for both samples was 45.

Second, using the subsample, ROC analysis was performed to determine a valid score standard for the ASQ:SE‐2/18 that can effectively distinguish between children with social, emotional and behavioral problems and those with normative development, using the dichotomous variable from the CBCL 1.5–5 as the criterion. On the CBCL 1.5–5, 13 children were identified at risk for either internalizing or externalizing problems. The ROC curves determined that the ASQ:SE‐2/18 had a good discriminatory power, AUC value was .85. The optimal balance between sensitivity and specificity on the ASQ:SE2/18 was observed at a cut‐off score of 40, yielding a sensitivity of 84.6% and a specificity of 73.6%. In contrast, the cut‐off score of 45 resulted in a lower sensitivity of 69.2% but a higher specificity of 80.5%. The ROC curve revealed that 26.0% of children scored above cut‐off value ≥40 (or 24.0% when the cut‐off value is ≥45) on the ASQ:SE‐2/18.

To assess convergence evidence, we examined how accurately the test can differentiate between individuals being at risk and individuals typically developing. We compared children's classification on ASQ:SE‐2/18 with those from the CBCL 1.5–5. The outcome was categorized as follows: (1) true positive: problem or risk on both tools; (2) true negative: no problem or risk on either tools; (3) false positive: risk on ASQ:SE‐2/18, but no problem or risk on CBCL 1.5–5; (4) false negative: no risk on ASQ:SE‐2/18, but risk or problem on CBCL 1.5–5 (see Table 3). Using a cut‐off score of 40, 84.6% (*n* = 11) of children identified as having problems based on the CBCL 1.5–5 would be correctly detected, compared to 69.2% (*n* = 9) with a cut‐off score of 45. However, at the cut‐off score of 40, 26.4% (*n* = 23) of children without problems would be misclassified as having difficulties, whereas this proportion decreases to 19.5% (*n* = 17) when the cut‐off score is set at 45.

**TABLE 3:** Contingency table showing agreement between ASQ:SE‐2/18 classification and criterion measure classification (CBCL 1.5–5).

		Criterion classification: CBCL 1.5–5	
		At risk (*n* = 13)	No risk (*n* = 87)
Screening classification: ASQ: SE‐2/18 40 (45) cut‐off	At risk	11 (9)	23 (17)
	No risk	2 (4)	64 (70)

Finally, we assessed the interrelations between the ASQ:SE‐2/18 total score and CBCL 1.5–5 scales using Spearman's correlation (Table 4). The ASQ:SE‐2/18 total score demonstrated moderate correlations with all CBCL 1.5–5 scales.

**TABLE 4:** Spearman's rho correlation coefficients between ASQ:SE‐2 total score and CBCL 1.5–5 scales.

ASQ:SE‐2/18	CBCL 1.5–5 at 18 months		
	Internalizing	Externalizing	Total score
Total score	.41**	.46**	.49**

*p < .05,.

**p < .001.

### 3.5 Discussion

The main aim of this study was to investigate the appropriateness of the Hungarian translation of ASQ:SE‐2/18 by assessing its internal consistency and convergent validity. As a result of Study 1, the Hungarian translation of the ASQ:SE‐2/18 was found to be culturally and linguistically appropriate. This finding was supported by feedback from a parent focus group. The Hungarian version was adapted with minimal changes, preserving the content and structure of the original questionnaire. The internal consistency was acceptable (Cronbach's *α* = .68 for the total sample, .77 for the subsample).

We examined evidence of convergence using CBCL 1.5–5 scales in a small sample (*n* = 100) including preterm children. The relationships between the ASQ:SE‐2/18 total score and the CBCL 1.5–5 scales showed moderate correlations. The correlation was similar to other international reports using CBCL 1.5–5 for validity test (Agarwal, et al., 2024; Alvarez‐Nuñez, et al., 2020; Stensen et al., 2018) in children aged 24 months.

To test the diagnostic accuracy of ASQ:SE‐2/18, a tentative cut‐off score was determined using two methods. According to the semi‐interquartile range (SIQR) method, a cut‐off score of 45 would be considered optimal, whereas the ROC analysis suggests a lower threshold of 40. We found that both cut‐off scores provide acceptable trade‐offs, however, applying the lower cut‐off score of 40 to the total sample resulted in 23.4% of children being classified as at risk, compared to 17.7% when using the cut‐off score of 45. The two cut‐off scores differed in terms of overall classification, 40 produced higher sensitivity (84.6%), but lower specificity (73.6%), whereas the score of 45 showed the opposite pattern, with reduced sensitivity (69.2%) but improved specificity (80.5%). Based on both the SIQR and ROC methods, a cut‐off score of 45 may be appropriate. The recommended cut‐off score, however, is determined in Study 2.

## 4 STUDY 2

### 4.1 The aim of Study 2

In Study 2, we conducted psychometric analyses of the ASQ:SE‐2/18 on a large representative sample to examine its dimensionality, to determine the recommended Hungarian cut‐off, and to evaluate the evidence of predictive convergence of the test. Study 2 used data from the Cohort ‘18 Growing Up in Hungary study (Veroszta et al., 2020), referred to henceforth as the Cohort ’18 study. The Cohort ’18 study is a longitudinal, countrywide and multidisciplinary birth cohort study, that applied the ASQ:SE‐2 to assess early socio‐emotional development risks of children in one of its data collection phases when the children were 18 months old. Our adaptation and psychometric analyses were thus limited to a single version of the ASQ:SE‐2, the 18‐month questionnaire.

### 4.2 Method

#### 4.2.1 Participants

Participants of Study 2 were drawn from the Cohort’18 study (Veroszta et al., 2020), a longitudinal survey initiated by the Hungarian Demographic Research Institute (HDRI) of the Hungarian Central Statistical Office. The study comprised five data collection phases until the children reached the age of three. These data collection phases will hereafter be referred to as “waves.”

Observation in Wave 1 began with 8287 pregnant women with an expected due date between April 1, 2018 and April 30, 2019. A complex multi‐stage sampling procedure was implemented to ensure representativeness (Kapitány, 2018). The primary sampling units were territorial health‐visitor districts, given that in Hungary, utilization of this service is obligatory for pregnant women. In the selected districts, all pregnant women were invited to participate. Consequently, the prenatal database was representative of the population of women who gave birth in Hungary in 2018, as evidenced by participants’ education, parity, official marital status, age and development indicator of the place of their residence (Szabó et al., 2021).

A total of 4918 children (51.9% males) whose mothers (or, in a few cases, grandparents, foster parents, or other primary caregivers) participated in the Wave 3 data collection—when children were 18 months old—were included in this study. Only children aged between 15 months 0 days and 20 months 31 days (corrected for prematurity) at the time of data collection were included. In the case of multiple pregnancies, data for the first‐born child was collected and analyzed. See Table 5 for sample characteristics.

**TABLE 5:** Descriptive characteristics of the analytical sample (*n* = 4918).

	*M*	SD
Age in months	18.2	.8
	n	%
Sex^t2^		
Male	2551	51.9
Female	2367	48.1
Respondent^t3^		
Biological mother	4903	99.7
Other primary caregiver	15	.3
Mother's number of children^t3^		
1	2321	47.2
2	1600	32.5
3	674	13.7
4 or more	308	6.3
Not applicable	15	.3
Mother's age^t3^		
19 or younger	97	2.0
20–24	500	10.2
25–29	1110	22.6
30–34	1566	31.8
35–39	1151	23.4
40 or older	479	9.7
Not applicable	15	.3
Moher's educational attainment^t1^		
Low	1355	27.6
Medium	1455	29.6
High	2089	42.5
Not applicable or missing	19	.4
Mother's relationship status^t3^		
Married	3172	64.5
Co‐habiting	1472	29.9
LAT or single	259	5.3
Not applicable	15	.3
Household's income quintile^t3^		
1. (lowest)	934	19.0
2.	945	19.2
3.	975	19.8
4.	885	18.0
5. (highest)	928	18.9
Missing value	251	5.1
Time of data collection^t3^		
Autumn 2019	612	12.4
Dec 2019–Jan 2020	1153	23.4
2020 Feb–Mar	937	19.1
Summer 2020	1358	27.6
Autumn 2020	843	17.1
Missing value	15	.3

LAT = couples living apart together.

^t1^ At Wave 1, during pregnancy.

^t2^ At Wave 2, among 6‐month‐olds.

^t3^ At Wave 3, among 18‐month‐olds.

*Note*: In cases where the respondent was not the biological mother, these descriptive data, with the exception of the sex of the child, are either missing or not analyzed in this research.

#### 4.2.2 Procedure

This study is based on data from the first, second, third and fifth waves of Cohort ’18. Recruitment of participants and data collection in the first two waves were carried out by local health visitors, following training by HDRI. These data collections took place in the third trimester of pregnancy and when the child was 6 months old, by either computer‐assisted or paper‐assisted personal interviewing (CAPI/PAPI), and by a self‐administered paper‐pencil questionnaire. Data collections in the third and fifth wave were carried out by professional interviewers via computer‐assisted personal interviewing (CAPI) and by a self‐administered online questionnaire, when the children were 18 and 36 months old.

The data collection of Wave 3 took place between October 2019 and November 2020 and was therefore affected by the COVID‐19 pandemic. Specifically, data collection was suspended between 18 March and 18 June in 2020, resulting in lack of data for this period.

Participation in the study was voluntary. All participants gave HDRI their written informed consent. The methodology of the research was in accordance with the Helsinki Declaration and the Code of Ethics of the Hungarian Psychological Association and was approved by an Ethical Committee (reference number 2022/1), set up for Cohort ’18, including professionals not included in the study group.

#### 4.2.3 Measures

##### 4.2.3.1 Sociodemographic characteristics

The sociodemographic characteristics analyzed were based on maternal reports across three waves of the study. The mother's highest completed level of education was assessed during pregnancy (Wave 1) and coded for the present analyses into three categories (i.e., low [ISCED 0–2], medium [ISCED 3–4], high [ISCED 5–8], based on the International Standard Classification of Education 2011 (UNESCO Institute for Statistics, 2012). The child's sex was assessed at 6 months of age (Wave 2). The characteristics analyzed from the time the child was 18 months old (Wave 3): mother's age (coded in six categories); mother's relationship status (i.e., married, cohabiting, living apart together or single); mother's household income (divided into net monthly equivalized household income quintiles, based on a square‐root equivalence scale, in accordance with OECD practice, calculated on the total sample); and mother's number of children (based on the number of living, biological children, coded in four categories). Due to the COVID‐19 pandemic, the time of data collection was also taken into account (coded in five periods).

##### 4.2.3.2 Ages and Stages Questionnaire: Social Emotional‐2

The 18‐month version of the Ages and Stages Questionnaire: Social Emotional‐2 (ASQ:SE‐2/18; Squires et al., 2015) was administered at Wave 3, when the children were 18 months old. Biological mothers completed this questionnaire as part of the self‐administered questionnaire; other primary caregivers responded verbally. See the Introduction and Study 1 for more information on the questionnaire and the Hungarian adaptation process.

##### 4.2.3.3 Strengths and Difficulties Questionnaire

The Strengths and Difficulties Questionnaire (SDQ) for 2–4‐year‐olds (Goodman, 1997; Hungarian adaptation: Birkás et al., 2008) was administered at Wave 5 with verbal responses, when the children were 3 years old. This 25‐item questionnaire consists of the following 5‐item subscales: emotional symptoms, conduct problems, hyperactivity/inattention, peer relationship problems, and prosocial behavior. Mothers rated the items on a 3‐point scale (0 = not true, 1 = somewhat true, 2 = certainly true). Missing values were replaced by the mean of the responses to the other questions in the same subscale, provided at least three items in the given subscale were answered, resulting in a response rate of 82%. Items representing internalizing problems (emotional symptoms and peer problems) and externalizing problems (conduct problems and hyperactivity/inattention) were summed to compute raw total scores for each (ranging from 0 to 20), and all items were summed to compute a total difficulty score (ranging from 0 to 40). Both the internalizing (Cronbach's *α* = .70) and externalizing (Cronbach's *α* = .70) subscales had a good internal consistency, as did the total score (Cronbach's *α* = .76). In addition to using continuous variables, a dichotomized dummy for risk of socio‐emotional difficulties was calculated. In the absence of population‐specific norms, this was based on the provisional banding of SDQ scores for 2–4‐year‐olds in the UK (available at sdqinfo.org). Children with high and very high scores were identified as being at risk, as opposed to those with close to average and slightly raised scores. Using this variable, 18.3% of children (*n* = 737, score ≥16) were identified as being at risk for socio‐emotional difficulties at the age of 3 years.

##### 4.2.3.4 Data analyses

Analyses were performed using SPSS 25 software, except for confirmatory factor analysis and internal consistency measures, which were performed using JASP 0.18.3.0.

For the confirmatory factor analysis of the ASQ:SE‐2/18, neither item 31, nor the concern responses were analyzed, following the method of Chen et al. (2016). Diagonally Weighted Least Squares (DWLS) was used as the estimation method. Missing values were not replaced (*n* = 4553). The models considered were: (1) the one‐factor solution, supporting the validity of the scoring schema of the total score (Squires et al., 2015), (2) the two‐factor solution based on Chen et al. (2016, 2020), (3) and a modified two‐factor solution that emerged from the present study after a re‐specification based on modification indices and theoretical justification. The fit of the models was evaluated on the basis of multiple indices, including *χ* ^2^, the comparative fit index (CFI), the Tucker‐Lewis index (TLI), the standardized root mean square residual (SRMR), and the root mean square error of approximation (RMSEA) with a 90% confidence interval (CI). Indicators of a close fit (CFI and TLI ≥.95, SRMR ≤.05; RMSEA ≤.06) and an acceptable fit (CFI and TLI ≥.90, SRMR ≤.08, RMSEA ≤.08) were determined (Hooper et al., 2008; Hu & Bentler, 1999). Internal consistencies were evaluated using Cronbach's alpha.

For further analyses, data replacement was performed according to the principles described in the ASQ:SE‐2 User's Guide (Squires et al., 2015). This means that for the total score, missing values were replaced by the mean of the responses to the other questions, provided there were no more than three missing items. For the 12‐item Emotional Difficulty subscale and the 18‐item Social Difficulty subscale, which were confirmed in the present study, missing values up to 1 and up to 2, respectively, were replaced by the mean of the responses to the other questions in the given subscale. The scores for expressing concern were also added to the total scores.

Descriptive statistics were computed for the sociodemographic characteristics of the sample, and for the scores of the total ASQ:SE‐2/18 and its subscales. Cut‐off points for the total score and the subscales were determined in the present study following the calculation method described by Squires et al. (2015) based on the semi‐interquartile ranges and medians. Socioeconomic and demographic correlates of being at risk and for having missing values on the ASQ:SE‐2/18 were analyzed using Chi‐squared tests, with Phi or Cramer‐V as a measure of effect size. Predictive validity was analyzed by bivariate Spearman's rho correlations between the continuous variables from the ASQ:SE‐2/18 and the SDQ, given that the distribution of variables deviated from the normal distribution. It was also assessed by a logistic regression model with dichotomized dummies based on these variables.

### 4.3 Results

#### 4.3.1 Factor structure and internal consistency

As can be seen in Table 6, while the RMSEA value for the one‐factor solution (Model 1D) indicated acceptable model fit, the other fit indices suggested unacceptable model fit. Model fit for the two‐factor solution (Model 2D‐SE) differentiating Social and Emotional Difficulties based on Chen et al. (2016, 2020) was close based on the RMSEA, acceptable based on the CFI and TLI values, while unacceptable based on the SRMR value.

**TABLE 6:** Fit indices of the confirmatory factor analyses of the 18‐month ASQ:SE‐2.

Model	*χ* ^2^	df	CFI	TLI	SRMR	RMSEA (90% CI)
1D	8255.92**	405	.779	.762	.144	.065 (.064; .066)
2D‐SE	2947.13**	404	.928	.923	.089	.037 (.036; .038)
2D‐CD	2212.85**	404	.949	.945	.069	.031 (.030; .033)

*Note*. **p < .001, 1D = one‐dimensional, 2D‐SE = two dimensional, Social and Emotional Difficulties, 2D‐CD = two dimensional, competencies and difficulties.

Inspection of the modification indices suggested an alternative solution. The two highest values indicated that items 15 (MI = 483.52) and 7 (MI = 389.40), which belong to the Emotional factor, would have a higher factor loading on the Social factor. Re‐classifying these two items resulted in a two‐factor solution (Model 2D‐CD), where one factor included reverse‐coded items, referring to socio‐emotional competencies, while the other included all the difficulty‐oriented items. Although this re‐specified model had the closest fit, it was decided to accept the solution proposed by Chen et al. (2016, 2020), who investigated several age ranges of ASQ:SE and also considered expert opinions. Factor loadings of the retained two‐factor solution are presented in Table 7.

**TABLE 7:** Factor loadings of the two‐factor model of the ASQ:SE‐2/18.

Factor	Indicator	Estimate	95 % CI	
			Lower	Upper
Emotional difficulty	Item 2	.53**	.51	.55
	Item 7	.35**	.32	.38
	Item 8	.57**	.55	.59
	Item 9	.64**	.62	.66
	Item 11	.57**	.55	.59
	Item 12	.78**	.76	.81
	Item 13	.60**	.58	.62
	Item 15	.49**	.45	.52
	Item 17	.60**	.58	.62
	Item 23	.88**	.86	.91
	Item 25	.70**	.67	.72
	Item 29	.38**	.36	.40
Social difficulty	Item 1	.62**	.58	.66
	Item 3	.63**	.59	.67
	Item 4	.35**	.32	.38
	Item 5	.22**	.19	.25
	Item 6	.42**	.39	.45
	Item 10	.77**	.73	.80
	Item 14	.55**	.52	.58
	Item 16	.76**	.72	.79
	Item 18	.57**	.54	.60
	Item 19	.73**	.70	.76
	Item 20	.73**	.69	.76
	Item 21	.48**	.45	.50
	Item 22	.58**	.55	.61
	Item 24	.52**	.49	.54
	Item 26	.71**	.68	.73
	Item 27	.83**	.79	.86
	Item 28	.51**	.49	.54
	Item 30	.79**	.76	.82

**p < .001.

Acceptable internal consistency was observed for the 12‐item Emotional Difficulty subscale (Cronbach's *α* = .74), and the 18‐item Social Difficulty subscale (Cronbach's *α* = .69), as well as for the total score (Cronbach's *α* = .73).

#### 4.3.2 Missing data

Overall, 92.3% of participants (*n* = 4538) responded to all items of the ASQ:SE‐2/18 questionnaire, while this rate was 96.0% for both the social difficulty (*n* = 4722) and emotional difficulty (*n* = 4719) subscales. Mothers with low household income, less than 25 years old and low educational attainment were slightly less likely to answer all items (see Table A1 in the Supplementary material). This was also true for unmarried mothers, mothers with four or more children, and mothers responding during the COVID‐19 virus outbreak in February and March 2020, but the effect size for these variables was negligible.

After data imputation, the total score was available for 99.3% (*n* = 4883) of respondents, which rate was 99.0% (*n* = 4867) for the Emotional and 99.8% (*n* = 4909) for the social difficulty subscale. Mothers younger than 19 years were most likely to have a missing total score after data imputation, with 3.1% having a missing value.

#### 4.3.3 Descriptive statistics and cut‐off scores

Descriptive data for the ASQ:SE‐2/18 for the Hungarian sample and the recommended cut‐off scores are presented in Table 8. The distribution of all variables deviated from the normal distribution. The two subscales showed a negligible correlation (*r*_s _= .16, *p* < .001). The calculated cut‐off point for the total score (>43.8) identified 21% of children as being at risk.

**TABLE 8:** Descriptive statistics of the ASQ:SE‐2/18 subscales and total score.

	Emotional difficulty (*n* = 4867)	Social difficulty (*n* = 4909)	Total score (*n* = 4883)
*M* (SD)	19.5 (17.7)	9.7 (13.0)	29.8 (24.7)
Median	15	5	25
Semi‐interquartile range	7.5	7.5	12.5
Range	0–110	0–180	0–200
90^th^ percentile	40	25	60
Skewness (se)	1.92 (.04)	3.89 (.04)	1.98 (.04)
Kurtosis (se)	5.10 (.07)	30.51 (.07)	6.00 (.07)
% FE	10.6%	34.5%	4.8%
Recommended cut‐off point^a^	26.3	16.3	43.8
Risk %	22.8%	17.8%	21.0%

% FE: Percentage of children who score zero on the given scale, indicating a floor effect.

^a^ Based on the semi‐interquartile method (adding 1.5 semi‐interquartile range to the medians).

#### 4.3.4 Socioeconomic and demographic correlates

Associations between socio‐economic and demographic characteristics and non‐optimal socio‐emotional development are presented in Table 9. In the following, the results for risk group ratios on the emotional and social difficulty subscales are summarized.

**TABLE 9:** Bivariate associations between socioeconomic and demographic characteristics and socio‐emotional risk rates.

Variables	Emotional risk		Social risk		Total risk	
	%	Test results	%	Test results	%	Test results
Child's sex^t2^						
Boy	23.9	*χ* ^2^(1) = 3.64, *p* = .056, Phi = −.03	20.7	*χ* ^2^(1) = 30.15, *p* < .001, Phi = −.08	22.9	*χ* ^2^(1) = 11.58, *p* = .001, Phi = −.05
Girl	21.6		14.7		19.0	
Mother's number of children^t3^						
1	21.5	*χ* ^2^(3) = 23.75, *p* < .001, Cramer's *p* = .07	17.9	*χ* ^2^(3) = 5.29, *p* = .152, Cramer's *V* = .03	20.0	*χ* ^2^(3) = 28.16, *p* < .001, Cramer's *V* = .08
2	21.7		16.6		19.7	
3	25.3		18.5		23.1	
4 or more	33.0		21.8		32.2	
Mother's age^t3^						
19 or younger	56.4	*χ* ^2^(5) = 260.35, *p* < .001, Cramer's *p* = .23	28.4	*χ* ^2^(5) = 16.93, *p* = .005, Cramer's *V* = .06	52.1	*χ* ^2^(5) = 235.22, *p* < .001, Cramer's *V* = .22
20–24	45.8		22.4		43.3	
25–29	24.8		17.6		20.9	
30–34	18.6		16.5		17.0	
35–39	15.8		17.1		15.9	
40 or older	18.7		17.3		18.6	
Mother's educational attainment^t1^						
Low	43.4	*χ* ^2^(2) = 451.50, *p* < .001, Cramer's *V* = .31	25.6	*χ* ^2^(2) = 87.06, *p* < .001, Cramer's *V* = .13	40.6	*χ* ^2^(2) = 447.64, *p* < .001, Cramer's *V* = .30
Medium	18.2		17.1		18.2	
High	12.9		13.2		10.6	
Mother's relationship status^t3^						
Married	18.3	*χ* ^2^(2) = 113.60, *p* < .001, Cramer's *V* = .15	15.4	*χ* ^2^(2) = 36.87, *p* < .001, Cramer's *V* = .09	16.7	*χ* ^2^(2) = 116.87, *p* < .001, Cramer's *V* = .16
Co‐habiting	29.8		22.1		27.6	
LAT or single	39.0		23.3		38.0	
Household's income quintile^t3^						
1. (lowest)	37.6	*χ* ^2^(4) = 203.94, *p* < .001, Cramer's *V* = .21	26.1	*χ* ^2^(4) = 65.84, *p* < .001, Cramer's *V* = .12	33.0	*χ* ^2^(4) = 184.58, *p* < .001, Cramer's *V* = .20
2.	28.4		19.6		28.3	
3.	20.1		16.5		18.6	
4.	15.9		13.8		14.7	
5. (highest)	13.2		13.8		11.4	
Time of data collection^t3^						
Autumn 2019	20.9	*χ* ^2^(4) = 5.89, *p* = .207, Cramer's *V* = .04	20.1	*χ* ^2^(4) = 2.66, *p* = .616, Cramer's *V* = .02	20.0	*χ* ^2^(4) = 2.82, *p* = .589, Cramer's *V* = .02
Dec 2019–Jan 2020	21.0		17.3		19.8	
2020 Feb–Mar	24.7		17.4		21.6	
Summer 2020	23.4		17.5		21.4	
Autumn 2020	23.8		17.8		22.5	

^t1^ At Wave 1, during pregnancy.

^t2^ At Wave 2, among 6‐month‐olds.

^t3^ At Wave 3, among 18‐month‐olds.

*Note*. Risk rates indicate the percentage of children in each group who scored above the cut‐off point—calculated using the semi‐interquartile method (adding 1.5 semi‐interquartile range to the median)—on the Emotional Difficulty subscale (Emotional risk), the Social Difficulty subscale (Social risk), or the total ASQ:SE‐2 scale (Total risk). LAT = couples living apart together.

The sex of the children showed significant, albeit negligible, associations with social risk, but no association with emotional risk. On the contrary, the mother's number of children only showed a significant and negligible association with emotional risk, which was highest for children of mothers who had raised at least four children. The time of data collection showed no significant correlation with any of the risk rates.

The remaining variables showed significant associations with both subscales, with stronger effect sizes for emotional risk (see Cramer's V values in Table 9). Children of mothers under 25 years of age showed the highest risk rate on both dimensions, while children of mothers aged 30 years or older had a lower‐than‐average rate of emotional risk. Regarding maternal educational attainment and household income, both the rate of emotional and social risk demonstrated a gradual decline as the background became more advantageous. In terms of maternal relationship status, children of married mothers had the lowest risk rate on both dimensions, while children raised by single mothers had a higher rate of emotional risk than those with a mother in a cohabiting relationship.

#### 4.3.5 Predictive validity

Socio‐emotional difficulties (based on SDQ) at the age of 3 were significantly and weakly correlated to socio‐emotional development at the age of 18 months, in terms of the ASQ:SE‐2/18 total and the Emotional Difficulty subscale, while negligible correlations were observed with the Social Difficulty subscale (see Table 10).

**TABLE 10:** Spearman's rho correlation coefficients between early childhood socio‐emotional development and difficulties.

ASQ:SE‐2/18	SDQ at 36 months		
	Internalizing	Externalizing	Total difficulties
Emotional difficulties	.20^**^	.30^**^	.32^**^
Social difficulties	.13^**^	.13^**^	.16^**^
Total score	.24^**^	.30^**^	.34^**^

** *p* < .001.

In a multivariate model, higher odds were confirmed for having socio‐emotional difficulties (indicated by high/very high SDQ total difficulty score) at the age of 3, for children characterized by emotional (OR = 3.55, 95% CI: 2.98, 4.22) or social (OR = 1.73, 95% CI: 1.42, 2.11) risk at the age of 18 months. The model was significant (*χ* ^2^(2) = 247.23, *p* < .001) and had a small explanatory power (Cox & Snell *R* ^2 ^= .06, Nagelkerke *R* ^2 ^= .10).

### 4.4 Discussion

The first objective of Study 2 was to investigate the dimensionality of the ASQ:SE‐2/18 using a large, representative sample. We compared the one‐factor (Squires et al., 2015) and two‐factor (Chen et al., 2016; 2020) solutions, with the two‐factor solution emerging as more appropriate. However, the CFA results also indicated an alternative model with even better fit indices. In this re‐specified structure, the coding of the items—non‐reversed or reversed—gave the organizing principle of the factors, hence they were called Competencies and Difficulties scales. In the Competencies scale, social‐emotional skills are all coded as reversed items (such as “Does your child let you know how he is feeling with gestures or words?”), while in the Difficulties scale, emotional regulation difficulties and behavioral problems (such as “Does your child hurt himself on purpose?”) are coded in non‐reversed. This type of item arrangement is somewhat consistent with the proposed dimensions by Chen et al. (2020), where at all age intervals the Social dimension has only items formulated in the direction of competence. On the other hand, the Emotional dimension contains only 2–3 competency‐related (reverse coded) items up to 24 months of age—there are 2 such item in the ASQ:SE‐2/18. At 30 months, however, there are already seven competency‐related items on the Emotional dimension. This finding aligns with the stronger correlation observed between the two sub‐factors from this age onwards (Chen et al., 2016, 2020), and with prior factor analytic results indicating that for children aged 30 months and older, the two‐factor fits less well than for younger groups (Chen et al., 2016)—or is even outperformed by a one‐factor solution (Alvarez‐Nuñez et al., 2020). Nevertheless, we decided to retain the model of Chen et al. (2016, 2020), that showed an acceptable fit as well, and to analyze Emotional and Social Difficulties in further analyses, since in this study we provided evidence for one age group only.

The subscales demonstrated acceptable internal consistency (Cronbach's *α* = .74 for emotional difficulty and .69 for social difficulty), negligible inter‐correlation, and remarkable floor effect, as well as a deviation from normal distribution. Cut‐off scores have been established for the total score (43.8), and for the two subscales, emotional difficulty (26.3) and social difficulty (16.3), identifying 21.0%, 22.8% and 17.8% of children as being at risk, respectively.

The findings indicate that sex differences in social‐emotional difficulties are either insignificant or negligible. However, a more disadvantageous socioeconomic and demographic background is associated with a greater risk of social and emotional problems, with more pronounced effects for emotional difficulties.

Evidence for the predictive validity of the ASQ:SE‐2/18 was provided by investigating its association with the 36‐month age SDQ data available in the Cohort’ 18 database. The 18‐month ASQ:SE‐2/18 total score and its emotional difficulty subscale exhibited a weak correlation with the SDQ scales. Utilizing a categorical approach, being at risk for both dimensions predicted being at risk for internalizing and externalizing problems at age 3, with a more pronounced effect observed for emotional difficulties.

## 5 GENERAL DISCUSSION

This research aimed to assess the validity and reliability of ASQ:SE‐2 for 18‐month‐old children through two consecutive studies on both non‐representative and representative Hungarian samples. The research focused on providing a culturally adjusted adaptation, evaluating its specificity and sensitivity, and finally investigating the dimensional structure of the ASQ:SE‐2/18, its socioeconomic and demographic correlates, and predictive validity.

The international adaptation of screening tools present challenges due to culturally diverse parental values, childrearing practices and parenting styles, especially when adjusted for non‐Euro‐American contexts (e.g. Chen et al., 2019). However, in the case of the Hungarian adaptation, our experience indicates that, with only few exceptions, the items of the questionnaire were well understood and appropriately aligned with Hungarian context of childrearing practices and the child‐parent relationship.

The ASQ:SE‐2 uses empirically determined cut‐off values designed to refer children to professional care and to follow up on their status. Squires et al. (2015) recommend that the establishment of the cut‐off scores should be culturally contextualized and based on national samples. Our analyses suggested a tentative cut‐off score of 45 in Study 1 and a recommended cut‐off score of 43.8 in Study 2, applying the SIQR method on a representative sample. Study 1 also provided evidence of convergence for this value using the CBCL 1.5–5 questionnaire. This cut‐off point is considerably lower than those recommended for 18‐month‐olds in the U.S. (65, Squires et al., 2015) or Iran (60, Shariatpanahi et al., 2024). The detection rate of children at risk (21.0% in Study 2), however, was found to be comparable to the outcomes of the U.S. validation study (Squires et al., 2015, 23.3%) and former international adaptations of ASQ:SE/18 and ASQ:SE‐2/18 that adopted the same cut‐off calculation (Heo & Squires, 2012, 16.2%; Bian et al., 2017, 15.6%; Krijnen et al., 2021, 19.8%; Shariatpanahi et al., 2024, 23.1%). In our opinion, the correspondence between the Study 1 and Study 2 cut‐off scores, as well as the fact that the resulting risk prevalences are similar to the clinical prevalence rates reported in former studies (Gleason et al, 2010; Gustafsson et al., 2017), serve as further support for the validity of the Hungarian cut‐off.

Previous factor analytic investigations of the dimensionality of the ASQ:SE and the ASQ:SE‐2 showed conflicting results, supporting one or two dimensions across certain age groups (Alvarez‐Nuñez et al., 2020; Chen et al., 2016; Edenius et al., 2024). In this study, we followed the tradition of testing a two‐dimensional structure proposed by Chen et al. (2016). We compared this model with the one‐factor solution (Squires et al., 2015) then a re‐specified two‐factor solution emerged from the present study. Although the re‐specified model, differentiating competencies and difficulties, showed the best fit in this study, we decided to retain the model by Chen et al. (2016, 2020) and proceeded to analyze Emotional and Social Difficulties in subsequent analyses. This final two‐dimensional model demonstrated an acceptable fit, considering the obtained model fit indices and previous international results (Alvarez‐Nuñez et al., 2020; Chen et al., 2016). It should also be noted that in the Hungarian sample the correlation between the two factors was remarkably lower than that reported by Chen et al.’s (2020) research (.16 vs .46). It is possible that while emotional difficulties and social competencies are both considered to be components of social‐emotional development, behavioral problems and regulatory difficulties may be more distinct construct from social and emotional competencies than expected—at least during infancy and early toddlerhood.

The internal consistency of the Total, Emotional Difficulty and Social Difficulty scales were found to be acceptable, but lower than reported in the U.S. validation study (Squires et al., 2015). This finding is consistent with the results of recent adaptation studies (e.g., Velikonja et al., 2017) and aligns with the conclusions of previous studies (Alvarez‐Nuñez et al., 2020; Squires et al., 2015) that the reliability of the ASQ:SE‐2 is better for children 24 months and older. The results raise the question of whether further adjustments, especially in the younger age intervals, may be needed to improve the reliability of ASQ:SE‐2. One possibility is to use a reduced set of items (Edenius et al., 2024). However, since this is a screening tool, assessing certain clinically significant symptoms may yield more accurate results and greater clinical utility than creating a reduced set with higher internal consistency.

Similar to previous adaptation studies (e.g. Heo & Squires, 2012; Agarwal et al., 2024) the distribution of the total ASQ:SE and ASQ:SE‐2/18 score and the subscales of Social and Emotional Difficulties were slightly skewed and showed high kurtosis. Although the proportion of children scoring zero on the total ASQ:SE‐2/18 (4.8%) was lower than that reported by Alvarez‐Nuñez (2020) in their Uruguayan sample (12.3%), these results highlight the challenge of distinguishing subtle differences in‐low‐level problems, particularly in the domain of social development.

Comparing the total score of ASQ:SE‐2/18 Study 1 found no significant gender differences and Study 2 presented only a negligible but significantly higher social risk for boys. These results confirm previous findings that mostly underline the lack of gender differences in early emotional and social risks (Squires et al., 2004). This trend gradually becomes more pronounced as children age. It is likely that as children grow, they will be increasingly exposed to peer and adult social influences that will make their behavioral repertoire more gender diverse. On the other hand, with the advent of the intensification of parental gender stereotyping, boys are perceived as being at higher risk for social problem behavior (Zsolnai et al., 2012).

Children raised by young, economically and educationally disadvantaged and single parents showed higher social‐emotional risks than their counterpart peers from more advantaged backgrounds. This finding supports previous evidence indicating that disadvantaged socioeconomic and sociodemographic background status is associated with elevated levels of social‐emotional problems (Alvarez‐Nuñez et al., 2020; Vaezghasemi et al., 2023) and can be interpreted within the framework of the Family Stress Model (Masarik & Conger, 2017).

The extent to which early screening questionnaires can predict later adjustment difficulties is a crucial indicator of their validity, as successful early identification increases the likelihood that vulnerable children will be referred to early prevention and intervention services. Guided by this consideration, we tested the predictive validity of the ASQ:SE‐2/18. Weak correlations were observed between the 18‐month ASQ:SE‐2/18 scores and 36‐month psychological maladjustment scores (assessed by the SDQ). Furthermore, social or emotional risk at 18 months increased the odds of social‐emotional difficulties at age 3 by 1.7 to 3.6 times. These results are in line with the findings of Agarwal et al. (2024), who found moderate significant positive correlations between 24‐month ASQ:SE‐2 and 48‐month CBCL scores. It is noteworthy that the 18‐month Emotional difficulty score predicted the SDQ total difficulty score with a higher odds ratio. Consistent with previous research (e.g. Pihlaja et al., 2025) our findings suggest that during the transition from infancy to toddlerhood, emotional regulation and behavioral problems may serve as more effective early indicators of maladaptive developmental pathways, than the attainment of social‐emotional milestones.

## 6 LIMITATIONS OF THE STUDY

Although our study used both a small and a representative national sample and examined the validity and reliability of the Hungarian version of the ASQ:SE‐2/18 from several perspectives, there are limitations to this research. Firstly, we observed heavy‐tailed, positively skewed distribution and a strong floor effect of the total score—and the subscales as well. These might reduce variability and could affect the interpretation of the results. Second, limitations arise in relation to concurrent and predictive validity assessments. Although our aim with the sub‐sample of Study 1 was to test the specificity and sensitivity of the questionnaire on a larger group of vulnerable children, our sample still contained a relatively low proportion of children identified as being at risk for either internalizing or externalizing problems. This did not allow for a more detailed examination of criterion validity along the CBCL subscales. Another limitation of the validity measures is the use of other mother‐reported questionnaires (CBCL and SDQ) as reference points, and the absence of a Hungarian cut‐off value for the SDQ 2‐4. Thus, the cut point was determined based on the United Kingdom general population (sdqinfo.org). Third, a general limitation is that maternal reports of children's behavior are positively biased. This phenomenon is discussed in previous research with regard to ASQ:SE and other parental report methods on early socioemotional problems (Alvarez‐Nuñez et al., 2020; Pihlaja et al., 2025; Squires et al., 2002) and is also in line with our observation that Hungarian mothers tended to be generally more positive about their child's behavior in a questionnaire measuring infant temperament (Sándor et al., 2023). Fourth, last periods of data collection were carried out during the COVID‐19 pandemic (from March 2020 – until August 2020) and Kuehn et al. (2024) found higher rates of positive ASQ:SE‐2 screening in the pandemic cohorts compared to prepandemic cohorts of 12‐ and 18‐month‐old children. Although these outcomes were performed in a low‐income sample participating in a Nurse‐Family Partnership program it can be conceived that the exposure to COVID‐19 might affect our results as well. However, the risk rates from different data collection periods show that the pandemic effect is probably not present in our sample. Fifth, item 31 and the concern‐type parental responses were excluded from the factor analysis in Study 2. The rationale behind this decision is that item 31 is overly general to be classified as either a social or an emotional difficulty, while the concern‐type responses are not an inherent part of the Likert‐type response scale. However, these were included in total score calculations, in accordance with the original theoretical conception of the screener—leading to some discrepancy between the factor analysis and other analyses. Finally, similarly to other adaptation studies, we omitted the overall items from the analysis. While we acknowledge the clinical value of these items, the lack of standardized scoring prompted us to exclude them from this quantitative research.

## 7 IMPLICATIONS AND FUTURE DIRECTIONS

Overall, our results indicate that the Hungarian version of the ASQ:SE‐2/18 can be used with caution as a screening instrument for socio‐emotional development in the general population. In our view, the 12‐item Emotional Difficulty subscale may be more effective for screening early risk. This scale shows a smaller floor effect, its distribution is closer to normal, it is more sensitive to social disadvantages and is a stronger predictor of later emotional and behavioral problems. Since the validation of one age interval does not mean the full applicability of the ASQ:SE‐2 in other age ranges, future research should address the adaptation of more age ranges into Hungarian, and test validity on a larger sample, including a higher proportion of children at risk for social‐emotional problems. In addition, it would be valuable to further test the factor structure, considering the possibility that in the early period there is a more pronounced separation between competency‐directed and difficulty‐directed items. Measurement invariance across gender should also be tested. By further analysis of the factor structure future research may aim to create an adapted version of the ASQ:SE‐2/18 that is sensitive to both social and emotional difficulties and is suitable for use in health visitor screening.

## CONFLICT OF INTEREST STATEMENT

The authors declare that they have no known competing financial interests or personal relationships that could have appeared to influence the work reported in this article.

## Supporting information



Supporting Information

## Data Availability

The data that support the findings of Study 1. are available on request from the corresponding author. The data that support the findings of Study 2. are available on request from the Hungarian Demographic Research Institute (HDRI) of the Hungarian Central Statistical Office for researchers who meet the criteria for access to confidential data. See also: http://www.demografia.hu/en/birth‐cohort‐study “How to access data—for researchers”. The data are not publicly available due to privacy or ethical restrictions.
