# Supplementary material for: Psychometric properties of the Hungarian version of the ages and stages questionnaires: Social–emotional‐2 for 18‐month‐old children
Source: Infant Ment Health J. 2025 Oct 9;47(1):e70050. doi: 10.1002/imhj.70050 (PMC12719909; doi:10.1002/imhj.70050)
Supplement: Supplementary file 1 — Supporting Information [file IMHJ-47-0-s001.docx]

Supplementary Material

Appendix

**Table A.1.**

*Bivariate associations between socio-economic and demographic characteristics and rates of missing data on the ASQ:SE-2/18 (n=4918)*

| Variables | Rate of any missing data (%) | Test results |
| --- | --- | --- |
| Child’s sex^t2^ | | |
| Boy | 7.3 | χ²(1)=1.68, p=.196, Phi=.02 |
| Girl | 8.2 |  |
| Mother’s number of children^t3^ | | |
| 1 | 7.1 | χ²(3)=8.16, p=0.043, Cramer’s V=.04 |
| 2 | 7.4 |  |
| 3 | 8.8 |  |
| 4 or more | 11.4 |  |
| Mother’s age^t3^ | | |
| 19 or younger | 22.7 | χ²(5)=58.82, p<.001, Cramer’s V=.11 |
| 20-24 | 13.0 |  |
| 25-29 | 7.2 |  |
| 30-34 | 7.3 |  |
| 35-39 | 5.6 |  |
| 40 or older | 6.5 |  |
| Mother’s educational attainment^t1^ | | |
| Low | 13.3 | χ²(2)=84.39, p<.001, Cramer’s V=.13 |
| Medium | 6.3 |  |
| High | 5.0 |  |
| Mother’s relationship status^t3^ | | |
| Married | 6.4 | χ²(2)=22.83, p<.001, Cramer’s V=.07 |
| Co-habiting | 10.3 |  |
| LAT or single | 9.7 |  |
| Household’s income quintile^t3^ | | |
| 1. (lowest) | 12.5 | χ²(4)=54.12, p<.001, Cramer’s V=.11 |
| 2. | 8.9 |  |
| 3. | 6.2 |  |
| 4. | 5.1 |  |
| 5. (highest) | 5.1 |  |
| Time of data collection^t3^ | | |
| Autumn 2019 | 6.4 | χ²(4)=10.03, p=.040, Cramer’s V=.05 |
| Dec 2019 - Jan 2020 | 7.6 |  |
| 2020 Feb - Mar | 9.9 |  |
| Summer 2020 | 7.7 |  |
| Autumn 2020 | 6.4 |  |

*Note*. LAT=couples living apart together. ^t1^At Wave 1, during pregnancy. ^t2^At Wave 2, among 6-month-olds. ^t3^At Wave 3, among 18-month-olds.
